# Social isolation upregulates *takeout* expression in female *Drosophila melanogaster* to promote sucrose preference

**DOI:** 10.1371/journal.pgen.1011985

**Published:** 2026-08-10

**Authors:** Elisabeth A. Tawa, Lisa Schneper, Katherine I. Cho, Mindy Sim, Eve Schoeffler, Daniel A. Notterman

**Affiliations:** 1 Department of Molecular Biology, Princeton University, Princeton, United States of America; 2 Princeton Neuroscience Institute, Princeton University, Princeton, United States of America; Geisel School of Medicine at Dartmouth, UNITED STATES OF AMERICA

## Abstract

Previous research suggests that social isolation induces changes in gene expression that encode a starvation-like brain state and reduce sleep. However, the extent to which social isolation alters behaviors via sex-specific brain changes is unclear. Here, we use *Drosophila melanogaster* to explore sex differences in isolation-induced behavioral and transcriptomic changes. Male and female adult flies were isolated for seven days, and multiple behavioral sex-based differences were identified through tests of activity, motivation, aggression, and sugar preference. RNA-seq analysis also identified several candidate genes that were associated with sex differences in isolation-induced behavioral changes. *Takeout* (*to*) expression and sucrose selection were upregulated exclusively in females following social isolation. Following *to* knockdown in *to*-expressing cells, sucrose preference decreased in socially isolated females but increased in males. Overall, our results suggest that manipulating *to* expression influences sucrose choice in opposite directions between females and males following social isolation. It is possible that isolation-induced *to* overexpression in non-neuronal cells in the female head could contribute to sex differences in this behavior.

## Author summary

Chronic stress contributes to detrimental health effects, but our understanding of how stress induces sex differences in brain gene expression and behavior is incomplete. Here, we use a combination of behavioral testing, RNA sequencing, and genetic manipulations in *Drosophila melanogaster* to explore how social isolation reveals latent sex differences in gene expression and stress-relevant behaviors. We found the most pronounced sex differences in behaviors related to food choice and motivation. RNA profiling revealed isolated female-specific upregulation of over 100 genes, with many of them relating to reproduction and energy metabolism. We manipulated expression of the candidate gene *takeout (to)* and found that downregulating *to* in all *to*-expressing cells decreases sucrose preference in isolated females but increases it in isolated males. Our results suggest that within a chronic stress context, sex-specific effectors in the head may regulate gene expression related to feeding and macronutrient choice to ensure that females prioritize survival over reproduction. Learning more about this system in flies could provide insight into functionally analogous pathways in humans that may be dysregulated in female-biased stress-related disorders.

## Introduction

Social isolation has emerged in recent decades as a worldwide public health issue. The World Health Organization estimates that one in four older adults and five to fifteen percent of adolescents experience social isolation and loneliness globally [1], while the Centers for Disease Control and Prevention estimates that one in three adults experience these problems in the United States [2]. In humans, isolation increases the risk of early death and is associated with several diseases and psychiatric conditions, including cardiovascular disease, stroke, type 2 diabetes, depression, anxiety, suicidality, and dementia [1, 2]. Social isolation has also been found to enhance aggression, depression, and anxiety in humans undergoing solitary confinement [3]. Although there is suggestive evidence of sex differences in biological processes leading to differences in health outcomes between men and women experiencing social isolation [4, 5], the exact molecular and neurobiological mechanisms remain largely unknown. In *Drosophila melanogaster*, chronic social isolation has been shown to reduce sleep, increase feeding, and contribute to a transcriptomic profile indicative of a starvation-like brain state [6]. However, the cited study only examined the effects of chronic social isolation on activity and feeding in males.

Our goal was to develop a battery of behavioral tests and phenotypes that could be used to assess sex differences in the behavioral response to social isolation. We identified a set of fly behaviors that reflected sex-specific changes following seven days of social isolation. The sex differences in these phenotypes prompted us to perform RNA sequencing (RNA-seq) on the heads of male and female flies from socially isolated and group-housed conditions for target identification. Manipulations of one of the emerging target genes, *takeout (to*), suggest mechanistic relationships between isolation, sex, gene expression changes, and sucrose preference in the value-based feeding decision (VBFD) test. Overall, this study supports the use of *Drosophila melanogaster* as a valid non-mammalian model for elucidating the molecular mechanisms of social isolation-induced behavioral change, as well as sex differences in these mechanisms.

## Results

### Locomotion increases to a greater extent in males compared to females following social isolation

**Fig 1:**
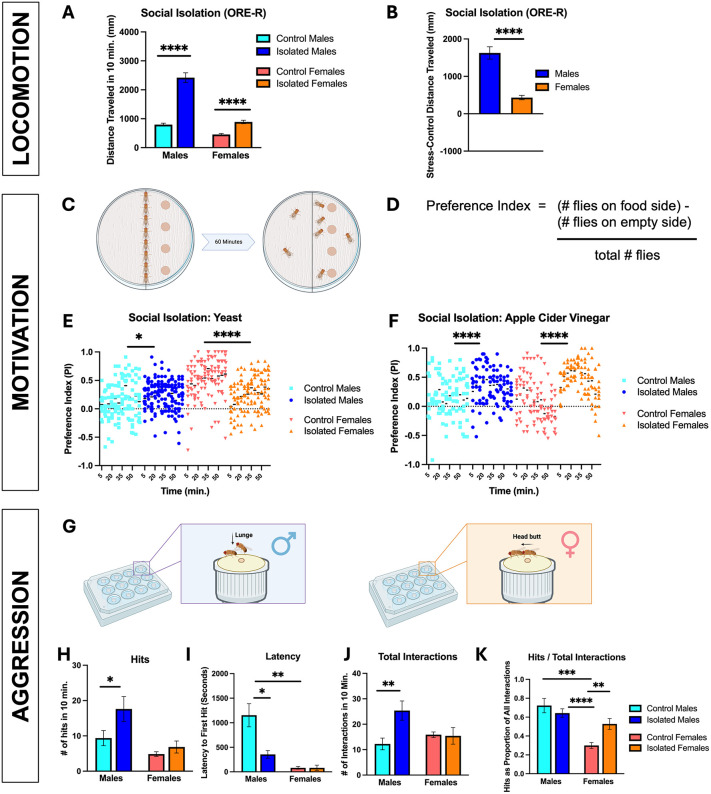
Social isolation differentially alters locomotor, motivated, and aggressive behavior between male and female flies. **A)** Flies that experienced social isolation (n = 99 individuals) traveled greater distances in a 10-minute period than controls (n = 100 individuals) for both males (U = 1729, p < 0.0001) and females (U = 2808, p < 0.0001). **B)** Taking differences in distance traveled between socially isolated flies and the control average for each sex revealed that the increase in locomotion in isolated males relative to control males was significantly greater (n = 99, U = 2850, p < 0.0001) than that observed between isolated females and control females (n = 99). **C)** Set up: flies were briefly anesthetized, positioned along the midline of the arena, and recorded during 60 minutes of exploration time. One half of the arena contained four dots of yeast paste or apple cider vinegar, and the other half had no food. Created in BioRender. Tawa, **E.** (2026) https://BioRender.com/sg414m7 **D)** A preference index (PI) was calculated at five-minute intervals, where PI = (the number of flies on the food side of the arena) - (the number of flies on the empty side of arena)/ the total number of flies. A value of 0 can be interpreted as at chance, due to equal numbers of flies being on each side. **E)** Isolated male flies (n = 13 groups, ~ 260 flies) are relatively more attracted to yeast paste than control males (n = 10 groups, ~ 200 flies) (U = 32, p = 0.02), yet isolated females (n = 11 groups, ~ 220 flies) are less attracted to yeast relative to control females (n = 10 groups, ~ 200 flies) (U = 8, p < 0.0001). **F)** Both isolated males (U = 4, p < 0.0001) and isolated females (U = 6, p < 0.0001) are more attracted to apple cider vinegar relative to their respective controls (n = 8 groups for all, ~ 160 flies). **G)** Set up: caps filled with standard fly food and topped with yeast paste were placed in a 12-well plate. Same-sex pairs of flies from the same experimental condition were aspirated into each well and recorded for 60 minutes. Male “hits” were defined as lunges and female “hits” were defined as head butts. Created in BioRender. Tawa, **E.** (2026) https://BioRender.com/aglrq9y. **H)** Socially isolated males (n = 16 pairs) hit more compared to group housed controls (n = 16 pairs, U = 76, p < 0.05). There was no difference in the number of hits between isolated (n = 15 pairs) and control females (n = 15 pairs, U = 101, p = 0.87). **I)** The latency between the flies’ first encounter on the food cup and the first hit was lower in socially isolated males relative to controls (U = 71, p < 0.03). Female controls had a significantly shorter latency to first hit compared to male controls (U = 40, p = 0.002) that did not differ from that of isolated females (U = 79, p = 0.57). **J)** Isolated males had a greater number of social interactions, aggressive or non-aggressive, than control males (U = 49.5, p = 0.002) in the 10 minutes following the first hit. **K)** Measuring the proportion of all social interactions that were hits, both control (U = 37, p = 0.0006) and isolated males (U = 14, p < 0.0001) had hits as a greater proportion of all social interactions relative to control females. However, social isolation significantly raised this proportion in females (U = 39.5, p = 0.002) to the levels of males. Error bars are ± SEM. *P < 0.05, **p < 0.01, ***p < 0.001, ****p < 0.0001.

To characterize social isolation as a valid fly stress model, we performed a series of behavioral tests that could capture changes of potential translational relevance. A walking assay revealed that social isolation increased distance traveled in both males (Fig 1A, *p* < 0.0001) and females (Fig 1A, *p* < 0.0001), but that the magnitude of the change was significantly greater in males (Fig 1B, *p* < 0.0001).

### Social isolation impacts motivation to seek food (yeast) differentially in males versus females

We next decided to assess whether social isolation differentially affected the goal-seeking behavior of males and females, specifically in the context of food motivation. To determine if social isolation alters motivated behavior in flies, we used two different stimuli, yeast paste and apple cider vinegar, to measure flies’ motivation to explore a physical space containing food. Yeast volatiles, either alone or as byproducts of fermenting fruit, attract *Drosophil*a [7–10]. Apple cider vinegar, through its high acetic acid content, mimics the odor of rotting fruit, which attracts flies as a source of food and mates. We developed a novel behavior test, inspired by an existing sucrose preference test [11], to assess whether social isolation alters the motivation of flies to investigate an attractive food or food odorant, and if so, whether sex differences are evident.

The preference index (PI) was calculated such that a value of 0 indicated no discrimination between the food side versus non-food side. Using a mixed effects analysis or three-way ANOVA (depending on whether the numbers across experimental groups were equal), we examined the effects of sex, social isolation, and the interaction of sex and social isolation on yeast preference over the 60-minute time course. There was a significant interaction between social isolation and sex (F(1, 477) = 50.89, p < 0.0001). Post-hoc tests indicated that social isolation increased males’ preference for yeast (Fig 1E, U = 32, p = 0.02) but decreased it in females (Fig 1E, U = 8, p < 0.0001). In contrast, using apple cider vinegar as an attractive odorant, we observed a significant effect of social isolation (F(1, 168) = 63.01, p < 0.0001) and the interaction of sex and social isolation (F(1, 168) = 9.14, p = 0.003), but not sex alone (F(1, 168) = 1.21, p = 0.27), on preference. These findings suggest that the interaction is largely driven by social isolation, and that for apple cider vinegar, both males (Fig 1F, U = 4, p < 0.0001) and females (Fig 1F, U = 6, p < 0.0001) were more attracted to the odorant side of the arena following social isolation relative to controls.

### Social isolation increases aggression in males and females across different measures

After testing flies on both hyperactivity and food motivation, we assessed aggression, a behavior that has been previously associated with social isolation in fly models [12–14]. We used a set up (Fig 1G) similar to one used by other research groups studying *Drosophila* aggression [15]: microtube caps were filled with standard fly food, topped with yeast paste, and placed in a 12-well plate, such that each well contained a single cap. This set up created an arena, where same-sex flies would encounter each other and begin fighting for territory and food. We defined aggressive “hits” in male flies as the lunge, a behavior in which the aggressor rears up on his hind legs and quickly snaps down on his opponent [15]. Aggressive “hits” in females were defined as the head butt, a movement in which the aggressor produces an abdominal thrusting-type motion and strikes the opponent with her head [16].

We found that social isolation increased the number of hits in males (p < 0.05) but not females (p = 0.87) (Fig 1H). Social isolation also decreased latency to first hit in males (p < 0.03) but not females (0.57), relative to the respective control groups (Fig 1I). Interestingly, females at baseline have a shorter latency to first hit compared to males. This is reflected in the significant difference in latency to first hit between male controls and female controls (p = 0.002). Thus, there may not be a difference between socially isolated females and control females in latency to first hit because the latency in control females is so short to begin with, creating a floor effect. Isolated males also showed a greater number of total social interactions (both aggressive and non-aggressive encounters) compared to controls (p = 0.002) which was not evident in females (Fig 1J). Finally, when examining aggressive hits as a proportion of all social interactions (Fig 1K), there is a difference between isolated females and their controls, such that hits make up a significantly greater proportion of social behaviors in isolated females (p = 0.002). This difference is not seen between isolated males and control males. Both control males (p = 0.0006) and isolated males (p < 0.0001) showed a higher hit proportion compared to control females, but isolated females’ increase in hit proportion rose to the extent that there was no difference between isolated females and isolated males. Thus, the increase in number of hits observed in isolated males may be a product of the fact that isolated males are also interacting more overall; there does not seem to be an increase in the frequency of hitting relative to other social behaviors. Isolated females’ increased proportion of hits out of total social interactions in combination with females’ overall short latency to hit may indicate enhanced female territoriality.

### Social isolation enhances sucrose preference in females

**Fig 2:**
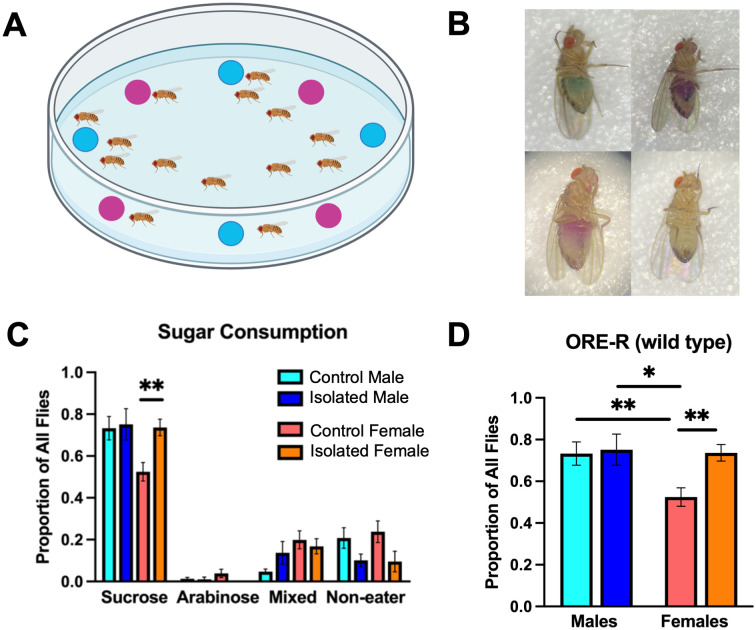
Social isolation increases sucrose preference in ORE-R females. **A)** Set up: flies were briefly anesthetized and allowed to explore an agarose plate with 2 different sugar solutions for 2 hours. Sucrose (150mM) was dyed blue, and arabinose (150mM) was dyed red. Created in BioRender. Tawa, **E.** (2026) https://BioRender.com/n8xc0ig. **B)** Flies were identified (clockwise from top left) as either blue, purple, none, or red and counted at the end of each 2-hour period. All flies shown are female, except for none (bottom right). **C)** Social isolation increased sucrose preference in females (t(15) = 3.14, p = 0.007). **D)** Control (n = 13 groups, t(22) = 2.85, p = 0.009) and isolated (n = 10 groups, t(19) = 2.67, p = 0.02) males both demonstrate a stronger sucrose preference than control females (n = 11 groups), but there are no significant differences between these groups and isolated females. Error bars are ± SEM. *p < 0.05, **p < 0.01.

After assessing changes in aggression, we employed the value-based feeding decision (VBFD) test as a final measure to evaluate the effects of social isolation on food motivation and choice. We followed an existing protocol [17], in which flies were placed in an arena for two hours to feed freely on either a blue solution of sucrose, a more nutritious sugar, or a red solution of arabinose, a less nutritious sugar (Fig 2A). The observed color (blue, red, purple, or none) of the crop was used to sort the flies into categories of sugar primarily consumed (Fig 2B). We performed additional testing to confirm that the flies did not have a color preference.

Following social isolation, the proportion of flies consuming sucrose increased significantly in females (Fig 2C, t(15) = 3.14, p = 0.007). No differences were observed in feeding choice between control males and isolated males. Focusing on sucrose, the nutritious sugar used in this test, we observed that both control males (Fig 2D, t(22) = 2.85, p = 0.009) and isolated males (Fig 2D, t(19) = 2.67, p = 0.02) had a greater proportion of flies consuming sucrose relative to control females, but that these differences were erased when comparing males to socially isolated females. Thus, isolation is associated with females increasing their sucrose preference to more closely resemble that of males.

### Social isolation uncovers sex differences across behaviors

The outcomes of all behavioral tests are summarized and compared by sex in Table 1. Overall, social isolation produced behavioral alterations across tests of activity, motivation, aggression, and sucrose preference. Following isolation, males increased their activity, their motivation to explore a space containing yeast paste or apple cider vinegar odorant, their number of aggressive hits (lunges), and overall social interactions in the aggression assay compared to male controls. Males’ latency to strike an opponent decreased, indicating heightened aggression. Females also increased their activity, motivation to explore the apple cider vinegar odorant, percentage of overall social interactions that were aggressive hits (head butts), and preference for sucrose relative to arabinose and no food. Isolated females decreased their exploration of the yeast paste side of the arena relative to control females.

**Table 1:** Summary of behavioral changes and sex differences following social isolation.

	Activity	Motivation		Aggression				Feeding Decision
	Walking	Yeast Pref.	ACV Pref.	Hits #	Latency	Social Interactions #	Hits %	VBFD % sucrose
Male								
Female								
								
								= INCREASED
								= NO CHANGE
								= DECREASED

### Social isolation does not impact lifespan

To explore the stress effects of social isolation further, we examined whether isolation affected longevity. We found no differences in survival between socially isolated and control flies for males (S1 Fig, χ^2^ = 2.71, df = 1, p = 0.10) or females (S1 Fig, χ^2^ = 0.25, df = 1, p = 0.62). Additional statistical tests, beyond the log-rank test, are detailed in Table 2.

**Table 2:** Statistical analysis of survival for social isolation.

MALES	Chi-square	df	P-value
Log-rank test	2.71	1	0.10
Gehan-Breslow-Wilcoxon test	2.45	1	0.12
Hazard ratio (Mantel-Haenszel): 0.68 (0.43, 1.08)			
Hazard ratio (log-rank): 0.76 (0.51, 1.12)			
**FEMALES**	**Chi-square**	**df**	**P-value**
Log-rank test	0.25	1	0.62
Gehan-Breslow-Wilcoxon test	0.006	1	0.94
Hazard ratio (Mantel-Haenszel): 1.11 (0.73, 1.71)			
Hazard ratio (log-rank): 1.10 (0.74, 1.65)			

### Differential expression analysis indicates female-specific overexpression of fat body-synthesized proteins following social isolation

**Fig 3:**
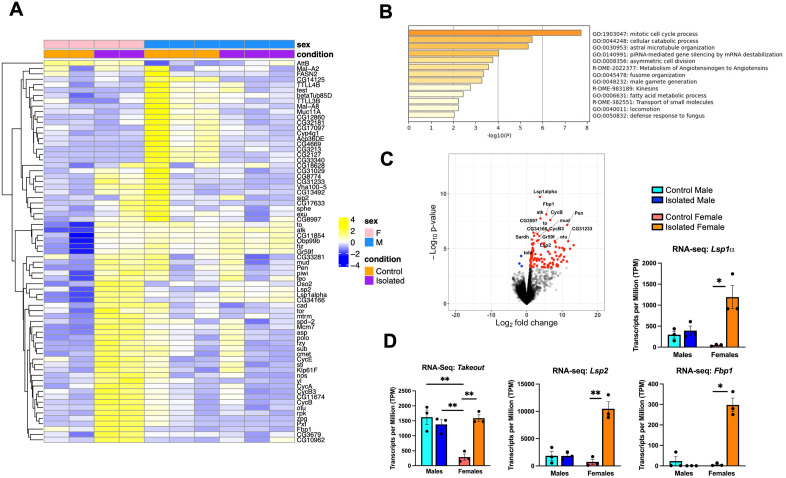
Socially isolated females demonstrate upregulation of multiple genes related to metabolic processes relative to control females and males. **A)** Heat map depicting the scaled (z-score transformed) log2 counts per million of genes differentially expressed (absolute value of log2-fold change > 2 and adjusted p-value < 0.05) when comparing the difference between SI female and control female with the difference between SI males and control males. **B)** Gene ontology (Metascape) of the significantly differentially expressed genes captured in the heat map reflects changes in broad categories of cell division and metabolism. **C)** The top differentially expressed genes (absolute value of log2-fold change > 1 and adjusted p-value < 0.05), when comparing the interaction between social isolation and sex, reflect upregulation in isolated females. The red points represent the genes upregulated in socially isolated females (relative to female controls) compared with socially isolated males (relative to male controls). In contrast, blue points represent the genes (*CG11211*, *VhaM9.7-a*, and *AttB*) upregulated in socially isolated males compared to the socially isolated females, relative to their respective controls. **D)** Gene expression, quantified in transcripts per million (TPM) from RNA-seq, indicates elevation in *takeout, Lsp2*, *Fbp1,* and *Lsp1α* in isolated females relative to control females that is not seen in males. At baseline, control females had lower TPMs compared to control males (t(4) = 5.07, p = 0.007) or isolated males (t(4) = 5.79, p = 0.004). However, socially isolated females had significantly higher TPMs compared to control females (t(4) = 8.56, p = 0.001) which are comparable to those of males. TPMs were also elevated following social isolation in females for *Lsp2* (t(4) = 7.06, p = 0.002), *Fbp1* (t(2.06) = 8.64, p = 0.01 with Welch’s correction), and *Lsp1α* (t(4) = 4.14, p = 0.01). Closed circles represent values derived from individual RNA samples, which were extracted from 200 fly whole heads. Error bars are ± SEM. *p < 0.05, **p < 0.01.

Upon identifying multiple sex differences in fly aggression and food motivation following social isolation, we decided to perform RNA-seq to uncover any sex- and isolation-based transcriptional differences. RNA samples were extracted in triplicate from whole fly heads for each experimental group. After performing multidimensional scaling (MDS) (S2 Fig), one sample from each of the female groups was removed to improve target discovery accuracy. When comparing the interaction between social isolation and sex, clusters of genes emerged that were upregulated in isolated females compared to control females and males (Fig 3A). Upon analyzing these significant genes with Metascape [18], changes were identified in groups of genes related to cell division (e.g., mitotic cell cycle process, asymmetric cell division) and metabolism (e.g., cellular catabolic process, fatty acid metabolic process) (Fig 3B).

Additionally, entering the significantly differentially expressed genes (comparing the interaction of social isolation and sex) into the STRING database [19] returned a network map of potential functional protein-protein interactions among them (S3 Fig). Applying the Markov Cluster (MCL) Algorithm [20] to our data then parsed our genes into 21 potential functional clusters, many of which coincided with the Metascape gene ontology results. Clusters with the strongest functional enrichment included genes related to mitotic cell cycle (S3 Fig), female reproduction (S3 Fig), and hemocyanins/hexamerins and secreted storage proteins (S3 Fig). STRING functional enrichment analysis also reflected enrichment in mitotic cell cycle processes and cell division, egg activation, piRNA metabolic process, and hemocyanins/hexamerins (S4 Fig). Finally, the significant genes were implicated in *Drosophila* phenotypes of abnormal meiosis and female sterility (S4 Fig).

Finally, we used a significance cut-off of p_adj_ < 0.05 to visualize differentially expressed genes in a volcano plot (Fig 3C). Only three significant genes were downregulated when comparing the interaction of social isolation and sex, while 105 were upregulated. The most significantly differentially expressed genes, by adjusted p-value, are listed in Table 3, along with their log fold change values. These genes were all upregulated in isolated females compared to isolated males, when taking into account the differences between the isolated groups and their respective controls.

**Table 3:** Top differentially expressed genes (interaction of social isolation and sex).

Gene Symbol	Log Fold Change	P-value	Adj. P-value
Lsp1alpha	3.72	1.96E-10	2.04E-06
Fbp1	5.54	7.54E-09	3.93E-05
atk	3.87	1.79E-08	6.21E-05
CycB	6.63	2.47E-08	6.43E-05
Pen	11.44	6.94E-08	0.000145
mud	6.08	2.25E-07	0.000391
CG3597	1.92	3.54E-07	0.000527
to	2.99	4.59E-07	0.000598
CycB3	3.55	7.05E-07	0.000734
CG34166	2.07	6.90E-07	0.000734
Sardh	1.50	1.49E-06	0.00141
Lsp2	2.90	1.92E-06	0.00146
otu	7.92	2.03E-06	0.00146
Gr59f	3.25	1.70E-06	0.00146
CG31233	11.70	2.10E-06	0.00146

An analysis of expression quantity data (transcripts per million, TPM) of some of the top genes identified by our model confirmed isolated female-specific overexpression of RNA transcripts for *larval serum protein-2* (*Lsp-2*), *larval serum protein-1ɑ* (*Lsp-1ɑ*), *fat body protein 1* (*Fbp1*), and *takeout* (*to*). *Lsp-2, Lsp-1ɑ, Fbp1*, and *to* are all synthesized in fat body cells [21–24], with the first three genes functioning as amino acid storage proteins and *to* being implicated in feeding behavior. *To* expression is higher in control (Fig 3D, t(4) = 5.07, p = 0.007) and isolated males (Fig 3D, t(4) = 5.79, p = 0.004) relative to control females, but this male-female difference is abolished when females have undergone social isolation. *To* expression is significantly higher in isolated females compared to control females (Fig 3D, t(4) = 8.56, p = 0.001). Similarly, social isolation increases *Lsp-2* (Fig 3D, t(4) = 7.06, p = 0.002)*, Lsp-1ɑ* (Fig 3D, t(4) = 4.14, p = 0.01)*,* and *Fbp1* (Fig 3D, t(2.06) = 8.64, p = 0.01) expression relative to control levels in females but not in males.

To confirm the RNA-seq results, we used qPCR as a secondary method to validate the expression of *to* in male and female fly heads. Using remaining RNA from the same samples that were submitted for sequencing, we analyzed expression of *to* in isolated males and isolated females relative to their respective controls. The qPCR data corroborated the RNA-seq findings. Overexpression of *to* following social isolation was specific to females and differed significantly from the gene expression change between isolated and control males (S5 Fig, t(4) = 6.14, p = 0.004).

### qPCR of ORE-R brains suggests that social isolation increases expression of *takeout* in females but not males

Following RNA-seq profiling and validation, we identified *to* as a gene demonstrating female-specific overexpression in the head following social isolation. However, this finding raised the question of whether *to* expression was changing in the brain or elsewhere in the head. For instance, the sex difference in isolation-induced *to* expression could stem from transcriptional changes in the pericerebral fat body, where *to* has been established to be transcriptionally regulated in a sexually dimorphic manner, or in the antennae [23] or foregut [25]. To gain a better understanding of whether *to* expression is changing within or outside of the fly brain, the brains of additional male and female flies that had experienced either social isolation or group housing (control conditions) were isolated, and RNA was extracted. The subsequent qPCR experiment again reflected female-specific overexpression of *to* following isolation (S6 Fig, t(10) = 2.28, p < 0.05).

### Adult *Drosophila* express *to* primarily in sensory neurons, epithelial cells of the gastrointestinal tract, and antennal nerve glia

In addition to validating our bulk RNA-seq results with qPCR, we also compared our findings with existing data from *Drosophila melanogaster* RNA-seq atlases. RNA-seq data from the modENCODE project [26] indicate that adult male *Drosophila* have higher whole organism-wide expression of *to* compared to adult females, which is consistent with our data comparing males to control female heads and brains.

Based on data from the Fly Cell Atlas project [27], *to* was most highly expressed in sensory neurons, sensory organ cells, glial cells, and epithelial cells out of all adult cell types in the *Drosophila* body (S7 Fig). The RNA-seq atlas data also showed *to* to be expressed in multiple epithelial cell types throughout the digestive system (S7 Fig), including the cells of the crop, midgut, esophagus, hindgut, and enteroendocrine cells, which secrete hormones to regulate digestion, metabolic homeostasis, and feeding behavior [28]. Finally, in glial cells, *to* showed the most widespread expression in adult antennal nerve glia (S7 Fig), which ensheath olfactory receptor neurons and may modulate the neuronal response to olfactory stimuli [29].

Based on these expression patterns and the behaviors to which *to* has been linked in the literature, we decided to first manipulate *to* expression systemically. This allowed us to assess whether there was a relationship between overall *to* expression and sucrose preference before focusing on a specific cell or tissue type. Next, because we were able to replicate our whole head RNA-seq finding in isolated brains, and glial cells only constitute approximately 10% of cells in the *Drosophila* nervous system [30], we decided to manipulate *to* expression in postmitotic neurons.

### Knocking down *to* in *to*-expressing cells decreases sucrose preference in socially isolated females and increases it in socially isolated males

A complete summary of our manipulations and behavioral findings is provided in Table 4. Data on the number of groups tested and average group size across all genotypes and experimental groups can be found in Supporting Information (S1 Table). In order to manipulate the expression of *to* in *to*-expressing cells, we mated virgin female flies with GAL4 expressed under the control of a *to*-specific promoter (*to*-GAL4) to males with a UAS enhancer 1) inducing the generation of RNA interference (RNAi) specifically targeted to *to* mRNA (UAS-*to*^RNAi^), or 2) driving expression of *to* (UAS-*to*). We performed qPCR tests to validate that *to* expression was downregulated in *to*-GAL4 x UAS-*to*^RNAi^ flies relative to ORE-Rs and upregulated in *to*-GAL4 x UAS-*to* flies relative to ORE-Rs across experimental groups (S13 Fig). The *to*-GAL4 x UAS-*to*^RNAi^ offspring were tested in the VBFD test to assess the effect of *to* knockdown in *to*-expressing cells on the proportion of flies that chose to feed on sucrose, a more nutritious sugar, over arabinose, a less nutritious sugar. The “proportion of all flies” dependent measure represents the proportion of all flies in a test group that fed exclusively or almost exclusively on sucrose, compared to all other combinations of feeding choice (a sucrose-arabinose mixture, arabinose only, or neither).

**Table 4:** Summary of behavioral results in VBFD test across genetic manipulations and experimental groups.

Effect of manipulation on sucrose preference (yellow = up, grey = no change, blue = down)				
	Control Males	Isolated Males	Control Females	Isolated Females
to RNAi in to-expressing cells (to-GAL4 x UAS-toRNAi)				
to overexpression in to-expressing cells (to-GAL4 x UAS-to)				
toRNAi in neurons (elav-GAL4 x UAS-toRNAi)				
to overexpression in neurons (elav-GAL4 x UAS-to)				
traRNAi in neurons (elav-GAL4 x UAS-traRNAi)				

Control males, isolated males, control females, and isolated females of *to*-GAL4 x UAS-*to*^RNAi^ and each parent genotype were tested. We observed an increase in sucrose preference in isolated males compared to control males (S8 Fig, t(14) = 2.31, p = 0.04), but a decrease in sucrose preference in isolated females relative to female controls (S8 Fig, t(15) = 5.35, p < 0.0001). This male isolation-induced increase in sucrose choice is specific to the knockdown manipulation and is not evident across either of the parent genotypes or the wild type. Moreover, knocking down *to* (*to*-GAL4 x UAS-*to*^RNAi^) reverses the trend observed in isolated females relative to control females that is also observed in the UAS-*to*^RNAi^ and ORE-R flies.

**Fig 4:**
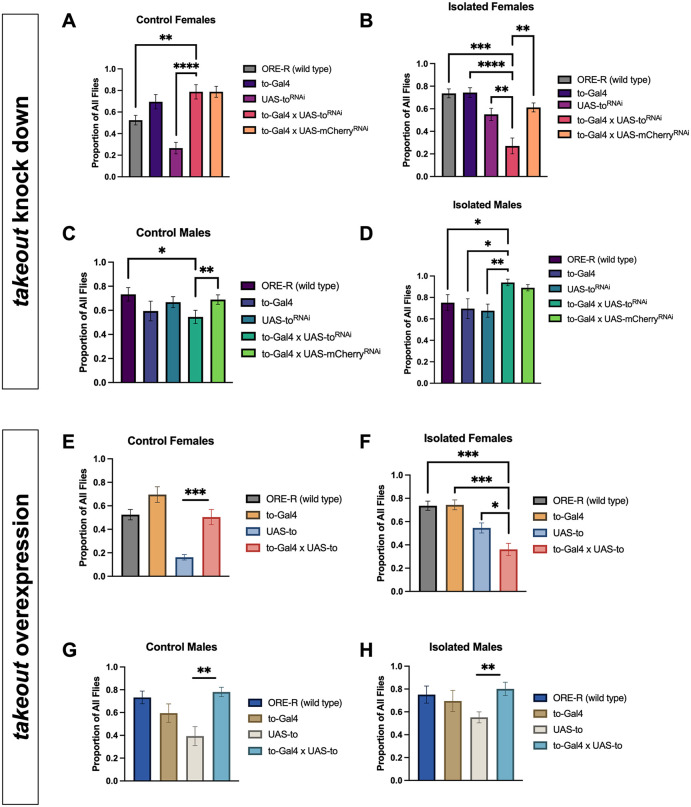
The proportion of all flies that primarily chose sucrose changes in opposite directions, relative to other genotypes, between isolated females and isolated males upon knock down of *to* in *to*-expressing cells. The proportion of all flies that primarily chose sucrose decreases relative to other genotypes in isolated females upon overexpression of *to* **in** *to***-expressing cells. A)** Sucrose preference is elevated in control females of the *to*-GAL4 x UAS-*to*^RNAi^ cross relative to ORE-R (t(18) = 3.41, p = 0.003) and UAS-*to*^RNAi^ (t(22) = 6.07, p < 0.0001) control females but does not differ from *to*-GAL4 or *to*-GAL4 x UAS-mCherry^RNAi^ control females. **B)** Sucrose preference is reduced in isolated females of the *to*-GAL4 x UAS-*to*^RNAi^ cross relative to isolated females of all other genotypes: ORE-R (t(12) = 5.24, p = 0.0002), *to*-GAL4 (t(13) = 5.53, p < 0.0001), UAS-*to*^RNAi^ (t(14) = 3.15, p = 0.007), and *to*-GAL4 x UAS-mCherry^RNAi^ (t(12) = 3.84, p = 0.002). **C)** Sucrose preference is reduced in control males of the *to*-GAL4 x UAS-*to*^RNAi^ cross relative to ORE-R control males (t(20) = 2.31, p = 0.03) and *to*-GAL4 x UAS-*mCherry*^RNAi^ control males (t(14) = 3.89, p = 0.002) but does not differ from parent genotype control males. **D)** Sucrose preference increases in isolated males of the *to*-GAL4 x UAS-*to*^RNAi^ cross relative to wild type and parent genotype isolated males: ORE-R (t(16) = 2.14, p < 0.05), *to*-GAL4 (t(15) = 2.39, p = 0.03), and UAS-*to*^RNAi^ (t(14) = 3.84, p = 0.002). **E)** Sucrose preference is elevated in control females of the *to*-GAL4 x UAS-*to* cross relative to UAS-*to* control females (t(16) = 4.50, p = 0.0004) but does not differ from ORE-R or *to*-GAL4 control females. **F)** Sucrose preference is reduced in isolated females of the *to*-GAL4 x UAS-*to* cross relative to isolated females of the wild type and parent genotypes: ORE-R (t(11) = 5.54, p = 0.0002), *to*-GAL4 (t(12) = 5.65, p = 0.0001), and UAS-*to* (t(13) = 2.74, p = 0.02). **G)** Sucrose preference is enhanced in control males of the *to*-GAL4 x UAS-*to* cross relative to UAS-*to* control males (t(18) = 3.91, p = 0.001) but does not differ from wild type or *to*-GAL4 control males. **H)** Sucrose preference is elevated in isolated males of the *to*-GAL4 x UAS-*to* cross relative to UAS-*to* isolated males (t(14) = 3.31, p = 0.005) but does not differ from wild type or *to*-GAL4 control males. *p < 0.05, **p < 0.01, ***p < 0.001, ****p < 0.0001.

Upon examining the effect of genotype on sucrose consumption, within a specific experimental group, we found that *to*-GAL4 x UAS-*to*^RNAi^ isolated females had a significantly *lower* proportion of sucrose-choosing flies compared to all other genotypes: ORE-R (Fig 4B, t(12) = 5.24, p = 0.0002), *to*-GAL4 (Fig 4B, t(13) = 5.53, p < 0.0001), UAS-*to*^RNAi^ (Fig 4B, t(14) = 3.15, p = 0.007), and *to*-GAL4 x UAS-mCherry^RNAi^ (Fig 4B, t(12) = 3.84, p = 0.002). In isolated males, the *to*-GAL4 x UAS-*to*^RNAi^ flies increased their proportion of sucrose preference relative to both parent genotypes (*to*-GAL4: Fig 4D, t(15) = 2.39, p = 0.03; UAS-*to*^RNAi^: Fig 4D, t(14) = 3.84, p = 0.002) and the ORE-Rs (Fig 4D, t(16) = 2.14, p < 0.05).

### Overexpressing *to* in *to*-expressing cells decreases sucrose preference in isolated females, but not males, relative to the ORE-R and parent genotypes

Upon testing the *to-*GAL4 x UAS-*to* flies on the VBFD test, we found that isolation did not seem to affect sucrose choice in either sex (males: S9 Fig, t(14) = 0.30, p = 0.77; females: S9 Fig, t(15) = 1.59, p = 0.13). However, in isolated females, sucrose preference was depressed in *to*-GAL4 x UAS-*to* flies compared to ORE-Rs (Fig 4F, t(11) = 5.54, p = 0.0002) and both parent genotypes (*to*-GAL4: Fig 4F, t(12) = 5.65, p = 0.0001; UAS-*to*: Fig 4F, t(13) = 2.74, p = 0.02). In both control (Fig 4G, t(18) = 3.91, p = 0.001) and isolated males (Fig 4H, t(14) = 3.31, p = 0.005), sucrose preference was increased in *to*-GAL4 x UAS-*to* flies compared to UAS-*to* males but did not differ from *to-*GAL4 or ORE-R flies of the same experimental condition.

### Driving to expression in neurons increases sucrose preference in males

After observing sex differences in sucrose preference upon *to* manipulation in all *to*-expressing cells (using the *to*-GAL4 driver), we proceeded to down- and upregulate expression of *to* in neurons alone. We began by mating *elav*-GAL4 virgin female flies with UAS-*to*^RNAi^ males to generate flies expressing RNAi targeted to *to* in postmitotic neurons. The *elav*-GAL4 line is commonly used in *Drosophila* research to drive the expression of GAL4 under control of the *elav* gene, which is active in postmitotic neurons throughout development and is expressed throughout most cells of the fly nervous system [31, 32]. Via qPCR, we validated that *to* expression was downregulated in *elav*-GAL4 x UAS-*to*^RNAi^ flies relative to ORE-Rs across experimental groups (S13 Fig).

**Fig 5:**
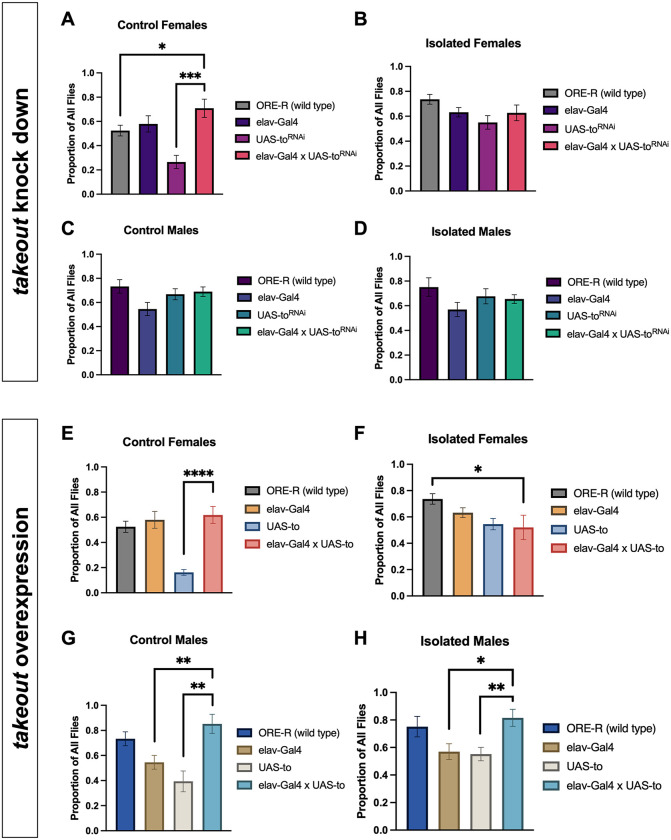
The proportion of all flies that primarily chose sucrose does not change in any experimental groups upon knock down of *to* in neurons. **The proportion of all flies that primarily chose sucrose increases relative to parent genotypes in control males and isolated males upon overexpression of *to* in neurons. A)** Sucrose preference is elevated in control females of the *elav*-GAL4 x UAS-to^RNAi^ cross relative to ORE-R (t(16) = 2.25, p = 0.04) and UAS-to^RNAi^ (t(20) = 4.72, p = 0.0001) control females but does not differ from *elav*-GAL4 control females. **B)** Sucrose preference does not differ between *elav*-GAL4 x UAS-to^RNAi^, wild type, and parent genotype isolated females. **C)** Sucrose preference does not differ between *elav*-GAL4 x UAS-to^RNAi^, wild type, and parent genotype control males. **D)** Sucrose preference does not differ between *elav*-GAL4 x UAS-to^RNAi^, wild type, and parent genotype isolated males. **E)** Sucrose preference is elevated in control females of the *elav*-GAL4 x UAS-to cross relative to UAS-to control females (t(12) = 7.15, p < 0.0001). **F)** Sucrose preference is reduced in isolated females of the *elav*-GAL4 x UAS-to cross relative to ORE-R isolated females (t(9) = 2.30, p < 0.05). **G)** Sucrose preference is enhanced in control males of the *elav*-GAL4 x UAS-to cross relative to UAS-to control males (t(15) = 3.63, p = 0.003) and *elav*-GAL4 control males (t(13) = 3.33, p = 0.005). **H)** Sucrose preference is also elevated in isolated males of the *elav*-GAL4 x UAS-to cross relative to UAS-to isolated males (t(12) = 3.30, p = 0.006) and *elav*-GAL4 isolated males (t(12) = 2.71, p = 0.02). *p < 0.05, **p < 0.01, ***p < 0.001, ****p < 0.0001.

We did not observe any differences in sucrose preference following social isolation in male or female offspring of the *elav*-GAL4 x UAS-*to*^RNAi^ cross (S10 Fig). An examination of within-experimental group effects of genotype on sucrose choice also failed to show an effect of *to* knock down in neurons on behavior. Although *elav*-GAL4 x UAS-*to*^RNAi^ control females increased sucrose preference compared to ORE-R (Fig 5A, t(16) = 2.25, p = 0.04) and UAS-*to*^RNAi^ (Fig 5A, t(20) = 4.72, p = 0.0001) control females, there was no difference relative to the other parent genotype, *elav*-GAL4. Across isolated females (Fig 5B), control males (Fig 5C), and isolated males (Fig 5D), the *elav*-GAL4 x UAS-*to*^RNAi^ flies did not differ in their sucrose preference from any other experimental control group (the wild type and parent genotypes) in a statistically significant manner.

Next, we set up *elav*-GAL4 x UAS-*to* crosses to assess the behavioral effect of *to* overexpression in neurons.When we tested the *elav*-GAL4 x UAS-*to* flies, there was not an effect of isolation on sucrose choice in females (S11 Fig, t(9) = 0.87, p = 0.41). For within-experimental group effects of genotype on sucrose choice, we noted that in males, both control flies (Fig 5G) and socially isolated flies (Fig 5H) increased sucrose preference relative to both parent genotypes. Thus, overexpressing *to* in postmitotic neurons seems to increase sucrose preference in males, but not females, irrespective of social housing or isolation.

### Masculinizing female neurons via RNAi targeted to *transformer (tra)* does not alter sucrose preference

Finally, we wanted to test whether altering the sexual identity of neurons to make female neurons more male-like affected sucrose choice. To do this, we crossed *elav*-GAL4 virgin females with UAS-*transformer* (*tra*)^RNAi^ males. This genetic manipulation has been used previously as a tool to masculinize neurons in female flies [33]. The UAS-*tra*^RNAi^ line uses double-stranded RNA to knock down *tra*, a gene in the *Drosophila* sex determination pathway that is crucial for female somatic sexual differentiation [34]. Using qPCR, we validated that *tra* expression was downregulated in *elav*-GAL4 x UAS-*tra*^RNAi^ flies relative to ORE-Rs across experimental groups (S13 Fig).

**Fig 6:**
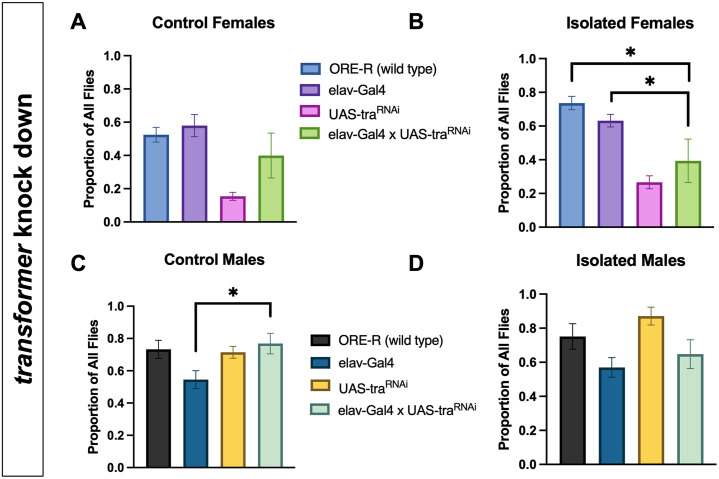
The proportion of all flies that primarily consumed sucrose does not differ between *elav*-GAL4 x UAS-*tra*^RNAi^ flies and other genotypes, across experimental groups. **A)** No significant differences were detected in sucrose preference between *elav*-GAL4 x UAS-*tra*^RNAi^ control females and control females of the wild type and parent genotypes. **B)** Sucrose preference is reduced in isolated females of the *elav*-GAL4 x UAS-*tra*^RNAi^ cross relative to ORE-R (t(10) = 2.54, p = 0.03) and *elav*-GAL4 isolated females (t(14) = 2.19, p < 0.05). **C)** Sucrose preference is elevated in *elav*-GAL4 x UAS-*tra*^RNAi^ control males relative to *elav*-GAL4 control males (t(13) = 2.61, p = 0.02). **D)** No significant differences were detected in sucrose preference between *elav*-GAL4 x UAS-*tra*^RNAi^ isolated males and isolated males of the wild type and parent genotypes. *p < 0.05.

Overall, we did not observe changes in sucrose preference in isolated males and isolated females relative to their respective controls in the *elav*-GAL4 x UAS-*tra*^RNAi^ offspring (S12 Fig, males: t(17) = 1.42, p = 0.17; females: t(17) = 0.91, p = 0.38). Within-condition comparisons across genotypes also did not reflect any clear differences in sucrose preference upon *tra* knock down in neurons. We did not see any differences across all genotypes in control females (Fig 6A) or isolated males (Fig 6D). Interestingly, qPCR validation revealed that knockdown of *tra* resulted in decreased expression of *to* across all experimental groups relative to ORE-R levels (S13 Fig). Since knocking down *to* expression in neurons through the *elav*-GAL4 x UAS-to^RNAi^ manipulation did not yield any changes in sucrose preference, it is consistent that decreasing *to* expression through neuron masculinization similarly did not produce clear changes in this behavior.

## Discussion

We first aimed to establish social isolation as a valid stressor in flies, using behavioral and survival data to capture stress-induced phenotypic changes. Overall, we found changes in males and females across behavioral tests of locomotion, food motivation, and aggression following seven days of social isolation. There were no significant effects of social isolation on lifespan in either males or females.

Our findings suggest a potential female shift in macronutrient preference from protein to sugar following social isolation. This is implicated by isolated females’ decreased exploration of the “yeast side” of the arena and increased preference for sucrose over arabinose. Isolated males were less discerning, showing increased exploration of both yeast and apple cider vinegar and no change in sucrose choice compared to controls. It could be evolutionarily advantageous for females to prioritize sugar over protein consumption while under the environmental stress of isolation, to favor survival over reproduction. Mated females have been shown to increase yeast consumption for the benefit of their offspring to the detriment of their own stress resistance and lifespan, suggesting a fitness-fecundity trade off [35].

We next focused on the sex-specific effects of social isolation on gene expression, and the potential stress-signaling pathways in which these genes participate. Using RNA-seq, we performed differential expression analysis and found effects of sex and social isolation on the expression of several genes related to metabolic processes and storage proteins. Specifically, we identified 105 genes that were significantly upregulated in isolated females compared with isolated males, which included genes encoding fat body-synthesized storage proteins and the related protein takeout (to). Ultimately, we focused on *to* as a target gene due to its previously characterized expression patterns and behavioral regulatory functions in the literature.

*To* was originally characterized as a gene encoding a secretory protein that is mainly expressed in the fly head [36] and mediates circadian feeding behavior [37]. It is also expressed in organs related to feeding and olfaction and is upregulated in these structures in response to starvation, with *to* mutants dying shortly following the onset of starvation [36]. Additionally, it has been found that *to* expression is regulated by sex-specific factors. In the pericerebral fat body surrounding the brain, the male-specific forms of Doublesex (Dsx^M^) and Fruitless (Fru^M^) proteins activate *to* expression, but the female-specific form of Doublesex (Dsx^F^) protein suppresses it [23]. This is consistent with our *to* TPM data from the male and female controls (Fig 3D). Moreover, *to* has been associated with male courtship behavior, which significantly decreases upon feminization of fat body cells via expression of the female Transformer protein (Tra^F^) in males [23, 38]. To the best of our knowledge, the effect of social isolation on *to* expression has not been studied.

Upon manipulating *to* expression with various GAL4-UAS combinations, we found that either up- or downregulating *to* in *to-*expressing cells decreases sucrose preference in socially isolated females. However, these same manipulations produce either increases or no changes in sucrose preference in socially isolated males. Taken together, these results suggest that *takeout* may function in isolated females like a neuromodulator; when either downregulated or upregulated in *to*-expressing cells, in the context of social isolation, sucrose preference is suppressed. The opposite behavioral response was observed in males.

When *to* expression was suppressed in neurons, we observed no effects. Based on these results, the sex-specific effects of *to* on sucrose preference following social isolation may be localized to non-neuronal cells within or in the periphery of the brain. It is possible that the isolated-female specific overexpression of *to* in brains from our qPCR analyses could be attributed to gene expression changes in glia or adipose cells that were not adequately removed from the brain tissue. In the future, it would be worthwhile to test whether modifying *to* expression in glial or fat body cells would replicate the results observed when *to* was manipulated in all to+ cells. Furthermore, it would be useful to repeat the *to* manipulation in neurons using alternative pan-neuronal GAL4 drivers to *elav*-GAL4, such as *nSyb*-GAL4 or *ChAT*-GAL4 [39], to strengthen our conclusion of a non-neuronal mechanism.

One possibility of how *to* could influence behavior is through the binding of juvenile hormone, a fly hormone that canonically regulates developmental transitions [40–42]. The protein encoded by *to* is highly structurally similar to juvenile hormone binding protein (JHBP), and it contains a putative binding domain for juvenile hormone [36, 37]. The secretion of takeout into the hemolymph from the pericerebral fat body and its binding of juvenile hormone could either hinder juvenile hormone signaling, by reducing levels of the hormone actively circulating, or facilitate juvenile hormone’s action on its targets via carrier protein function.

### Limitations and future directions

Although the present study has promise for elucidating the molecular mechanisms of sex-based responses to social stress, it is important to note its experimental and interpretational limitations. First, regarding the VBFD test, we quantified sucrose preference by categorizing the flies by crop color as primarily consuming sucrose, arabinose, both sugars (in roughly equal quantity), or nothing, and then calculating a proportion of sucrose-consuming flies out of all flies. The VBFD test does not provide absolute quantification of sucrose consumed, only preference for sucrose over arabinose. We also did not closely examine how flies were choosing. For example, for flies that consumed both sugars, we did not distinguish between flies that began eating arabinose and then switched to sucrose midway through the feeding period, or flies that were indiscriminately feeding between sucrose and arabinose during the entirety of the experiment. To gain a better understanding of how isolation might be influencing female feeding motivation and choice, it would be important to conduct a choice assay between protein and sugar. Future experiments incorporating macronutrient choice and using more rigorous approaches, such as the CApillary FEeder (CAFE) assay [43] to quantify food intake, are necessary to support the interpretation of an isolation-induced dietary switch in females.

Additionally, the present study did not examine sexual behavior or take sexual experience into account when considering the effects of social isolation on other types of behaviors. Tests of courtship and sexual receptivity could be added to examine how social isolation impacts male and female mating. These findings could inform whether isolation might influence female feeding preferences to shift resources away from reproduction. It should also be noted that reproductive state (e.g., virgin versus mated, or mated egg laid versus mated egg not laid) can strongly influence behavior, metabolism, and gene expression in *Drosophila* [44–47]. The added variability from reproductive state may account for why studies of social isolation have historically been conducted in male flies. In this study, we only tested mated females, but both virgin and mated females should be tested in the future to strengthen claims about the sex-specific effects of isolation on behavior.

Future experiments should also be conducted at more precise zeitgeber times (ZTs) to assess isolation phenotypes. Feeding, locomotion, and *to* expression are all strongly circadian-regulated [37]. However, the detection of behavioral differences despite testing across different circadian time points could indicate robustness of the observed phenotypic changes.

Regarding the transcriptional analyses, we validated our RNA-seq results by qPCR for selected genes. We used bulk RNA-seq, but performing single-cell RNA sequencing would be useful for determining if female-specific *to* overexpression is driven by a specific cell type.

Finally, our manipulations of *to* expression were limited in that they were broad and only focused on spatial control of gene expression. In our *to*–GAL4 x UAS-*to*^RNAi^ flies, we knocked down *to* in all *to*-expressing cells of the body, not just the ones in the head that were detected in our RNA profiling, and not with respect to developmental timing. For future experiments, it would be helpful to perform immunohistochemistry or fluorescence in situ hybridization to determine where *to* is being overexpressed in the isolated female head or brain and then target manipulations to a more specific cell or tissue type.

## Conclusion

Overall, chronic social isolation alters internal states and behaviors across animal species, and it may do so in a manner that differs by sex. We have found that social isolation in *Drosophila* impacts motivated behavior and sucrose choice differentially between males and females, and that these behavioral changes may depend on sex differences in the expression of the gene *to* in the fly’s head. One potential explanation for the increased expression of storage proteins and takeout following social isolation is that these changes allow female flies to tune their behavior to achieve an energetic balance appropriate for a specific environmental or social context. Despite the lack of an exact genetic ortholog for *to* in humans, it is also possible that takeout could serve as a functional analog to other mammalian proteins. For example, in mammals, lipoproteins in the brain and their metabolites have been found to regulate feeding behavior and energy balance [48]. Collectively, our findings support the use of *Drosophila melanogaster* as a model system for exploring female-specific molecular mechanisms related to stress-induced changes in food choice, energy metabolism, and possibly fertility.

## Materials and methods

### Fly stocks and rearing

All fly stocks were raised on standard medium (cornmeal/yeast/molasses/agar) at 25°C and 40% relative humidity on a fixed 12-h light/dark cycle. Male and female *Oregon-R* flies (wild type) and lines from the Bloomington *Drosophila* Stock Center (BDSC) were used for the majority of these experiments. Behavioral experiments were conducted within a ZT4-ZT8 time window. The following fly lines were obtained from the BDSC: *to-*GAL4 (#80938), *elav-*GAL4 (#8765), UAS*-to* (#81000), and UAS*-to*^RNAi^ (#55982). *Oregon-R* and UAS*-mCherry*^RNAi^ were gifts from Dr. Jodi Schottenfeld-Roames, Princeton University. The UAS*-tra*^RNAi^ (Vienna *Drosophila* Resource Center #2560, also known as traIR) stock was kindly provided by Dr. Rachel Monyak, Stonehill College.

### Experimental setups and design

#### Social isolation.

For the social isolation stress model, we used an experimental procedure for chronic social isolation already characterized in the literature [6]. Newly-eclosed male and female flies were housed together for three to five days to acquire social experience. Flies were then housed either singly or in same-sex groups of 25 for seven days. Within 24 hours of the final day of isolation or group housing, flies were either frozen on dry ice for RNA extraction, tested for behavior, or moved to/kept in same-sex group housing for survival analysis.

#### Survival.

To measure survival, flies (n = 50 per experimental group) underwent social isolation or group-housed control conditions for seven days, following the previously described protocol. At the end of the stress period, all flies were moved into group housing in standard food vials. The flies were counted once per day, and any deaths were recorded, along with the date of death, until all flies were deceased.

### Behavioral tests

#### Locomotion assay.

Flies were transferred to the testing chamber via aspiration or brief anesthesia with CO_2_. Upon analysis, we found that the use of CO_2_ did not alter activity when compared to aspiration. Each chamber consisted of a 55mm diameter petri dish filled with 1% agarose gel, to provide the flies with enough room to walk but not fly. The flies were put into same-sex groups of 10, recorded for 10 minutes, and frozen on dry ice. Videos were analyzed using EthoVision XT video tracking software (Noldus Information Technology, VA).

#### Motivated behavior tests.

Flies were tested in the arena previously described in “locomotion assay” to evaluate anhedonia-like behavior or discrimination of a food source. Flies were briefly anesthetized using CO_2_ and lined up along the midline of a filter paper evenly divided into two halves: one with yeast paste and one with nothing. Approximately 20 same-sex flies were tested in each group. Behavior was recorded for 60 minutes, and a preference index (PI) was calculated at five minute intervals as PI = [(# of flies on the yeast side) – (# of flies on the empty side)]/ (total). This test was repeated using apple cider vinegar in place of yeast paste, as an additional appealing food stimulus.

#### Aggression assay.

To test aggression, we modified an experimental protocol previously described by the Kravitz lab [15]. Testing occurred within 24 hours following the end of social isolation or crowding stress. Twelve-well plates were used as behavior chambers for testing same-sex, same-experimental condition pairs of flies. For group housed controls, flies in a fighting pair were taken from different vials to account for social novelty. To create an arena/territory, white plastic microtube caps were filled with standard fly food, topped with a dot of yeast paste, and placed in the wells. A transparent plastic covering with drilled holes was placed on top of the well plate, so that flies could be gently aspirated into the wells at the same time. The holes were then plugged with short screws. Fights were recorded for one hour using a Sony Digital Handycam camcorder (Sony Group Corporation, Tokyo, Japan).

Only behavior that took place in the arena was scored. Flies that did not encounter each other on the food cup within 45 minutes of recording were excluded from analysis. A goal sample size of n = 15 fighting pairs was decided prior to the start of experiments. For the fighting pairs, the times of the first encounter on the food cup and the first hit were recorded to calculate latency to initiate fighting. Fights were then scored for 10 minutes following the first hit (lunge for males or head butt for females). The male lunge was defined as a fly rearing up on its hind legs and snapping downwards onto its opponent. The female head butt was defined as a fly thrusting forward horizontally and then recoiling from the opponent. Number of hits, latency, number of overall encounters, and hits as a percentage of all encounters were used as measures to quantify and interpret aggressive behavior.

#### Value-based feeding decision (VBFD) test.

The value-based feeding decision (VBFD) test [17] was used as a two-choice assay to examine feeding decision making. Flies were briefly anesthetized with CO_2_ and placed in the center of the arena used for the locomotion and motivated behavior tests, a 55 mm diameter petri dish filled with 1% agarose gel and topped with a layer of parafilm. Sugars were dissolved in plain water to make two solutions: a 150 mM sucrose solution and a 150 mM arabinose solution. Each solution was labeled with a blue dye (0.01% erioglaucine disodium salt, Acros Organics, Cat# 229730250) or red dye (0.1% Food Red No. 106, TCI, Cat# F0143). Alternating droplets (20 μL volume) of each liquid food were placed evenly around the perimeter of the dish, such that there were eight droplets total, four of each solution. Flies were allowed to explore the arena and feed in the dark for two hours. At the end of this period, flies were collected and counted under a microscope. They were scored by color: blue (ingested primarily blue food), red (ingested primarily red food), purple (ingested both foods), or non-eater (no color accumulation in the abdomen).

### RNA extraction

For each sample, flies were decapitated on dry ice. RNA was extracted and purified from homogenized whole heads using a phenol chloroform organic extraction. Samples were also treated using either the RQ1 RNase-Free DNase kit (Promega Corporation) or the TURBO DNA-*free* Kit (Invitrogen) to address genomic DNA contamination. Total RNA samples were sequenced by the Princeton Genomics Core or used for downstream applications. The samples submitted for RNA-seq were extracted from 200 whole heads. To obtain brains, 20–40 *Drosophila* heads were dissected one at a time in cold Schneider’s medium. Brains were transferred to a microtube on ice containing 200 μL of Schneider’s medium, briefly spun down on a benchtop centrifuge, and then frozen at -80°C. Prior to homogenization in TRIzol (Invitrogen), samples were spun down, and the Schneider’s medium covering the brains was removed.

### Quantitative Polymerase Chain Reaction (qPCR)

RNA was reverse transcribed to complementary DNA (cDNA) using the SuperScript III First-Strand Synthesis System (Invitrogen). Random primers and a 200 ng total RNA template were used in a 20 μL reaction, following the instructions.

For real-time qPCRs, samples used SYBR Green PCR Master Mix (Qiagen or Applied Biosystems, Thermo Fisher Scientific). The PCR was performed in a 10 μL reaction including 3 μL of cDNA and 7 μL of master mix. The master mix for each gene consisted of 0.5 μL of each primer (10 μM), 5 μL SYBR Green, and 1 μL RNase-free water. Forward and reverse primer sequences for all genes can be found in Supporting Information (S2 Table). We used the following PCR conditions: initial incubation at 95°C for 15 min, followed by 40 cycles of 95°C for 15 s and 60°C for 1 min. Relative quantification was performed using the 2^−△△CT^ method [49].

### Transcriptome analysis by RNA Sequencing (RNA-Seq)

#### Sequencing.

RNA-seq was performed by the Princeton University Genomics Core Facility. The integrity of total RNA samples was assessed on the 2100 Bioanalyzer system using the RNA 6000 Pico Chip (Agilent Technologies, CA). The poly-A containing RNA transcripts were enriched from one microgram of total RNA for each sample using oligo-dT beads, further fragmented, and converted to cDNA and Illumina sequencing libraries using the PrepX RNA-seq library preparation protocol on the Apollo 324 NGS Library Prep System (Takara Bio, CA). Different DNA barcodes were attached to each library. The RNA-seq libraries were examined on Agilent Bioanalyzer DNA High Sensitivity chips for size distribution, quantified by Qubit fluorometer (Invitrogen, CA), and pooled at equal molar amounts. Each library pool was denatured and sequenced on one Illumina NovaSeq 6000 S Prime lane using the 100 cycle v1.5 kit. Raw sequencing reads were filtered by Illumina NovaSeq Control software, and only the Pass-Filter (PF) reads were used for further analysis.

#### Analysis pipeline.

Sequences were aligned to the *Drosophila melanogaster* genome (BDGP.46) [50,51] using STAR (v. 2.7.10b) [52]. Duplicates were removed using the Picard Toolkit (v. 2.27.5) [53] MarkDuplicates function. The number of reads mapping to each gene was determined using htseq-count (HTSeq v. 2.0.5) [54]. Differential gene expression analysis was performed in R (v. 4.4.0). All read counts from rRNA and pseudo-rRNA genes were removed. Normalization was performed using edgeR (v. 4.4.1) [55]. Multidimensional scaling (MDS) plots were used to identify outlier samples, which were then removed from the analysis. Data was prepared for linear modeling with limma (v. 3.60.6) [56] using voom [57]. The design matrix was constructed to model the relationship between gene expression and condition (Female-SI, Female-Control, Male-SI, Male-Control) adjusted for batch. Contrasts were used to compare gene expression differences between the following groups: 1. SI and control females, 2. SI and control males, 3. SI vs controls ((SI Female + SI Male)/(Control Female + Control Male). A fourth contrast was included to compare the difference between SI Female and Control Female with the difference between SI Male and Control Male. Differentially expressed genes were filtered based on an FDR-adjusted p-value of < 0.05 to determine significant genes with positive or negative values of log2FoldChange when comparing socially isolated males to control males, socially isolated females to control females, and the interaction between social isolation and sex. Heatmaps and additional plots were created using the R packages ComplexHeatmap (v. 2.21.2) [58] and ggplot2 (v. 3.5.1) [59].

### Statistical analysis

Statistical analyses were performed using GraphPad Prism 10.4.0 software (GraphPad Software, Inc., La Jolla, CA). The Shapiro-Wilk test of normality (passed at *p* > 0.05) was used to determine whether data followed a parametric distribution. Unpaired t-tests were used for parametric data assuming equal variances, and Welch’s t-tests were used if the samples had unequal variances based on the result of the F-test. If the data did not pass the test for normality distribution, nonparametric testing was used. All nonparametric data were analyzed with the Mann–Whitney *U* test for between-group comparisons. Effects were deemed statistically significant at *p* < 0.05. The Grubbs’ test was applied to identify and exclude outliers in the data. Log-rank tests were used to determine differences in survival curves, with *p* < 0.05 deemed statistically significant. A mixed-effects model (restricted maximum likelihood) or 3-way ANOVA were used to compare differences between stressed and control flies’ preferences for yeast or apple cider vinegar, respectively, between males and females over time. All statistics for two-way (S3 Table) and one-way (S4 Table) ANOVAs are provided in Supporting Information.

## Supporting information

S1 Fig Social isolation does not affect lifespan in male or female flies. Log-rank tests showed no significant differences in survival between isolated males (n = 50) and group-housed control males (n = 50, χ² = 2.71, df = 1, p = 0.10) or isolated females (n = 50) and group-housed control females (n = 50, χ² = 0.25, df = 1, p = 0.62).
(TIFF)

S2 Fig Multidimensional Scaling reflects similarity and dissimilarity within and between experimental groups. One sample (that was most dissimilar from the other two) was removed from each of the female groups to improve target discovery. Key: open = control, solid = social isolation; blue = male, orange = female; square = batch 1, circle = batch 2.
(TIFF)

S3 Fig Significant differentially expressed genes, when comparing the interaction between social isolation and sex, are connected in broader networks of protein interactions. A) All connected interactions are depicted, with non-connected genes removed. We specified a setting of “medium interaction strength” in STRING. The thickness of the connecting line between genes roughly corresponds to the strength of data support for gene interaction. B) One of our clusters that showed the strongest functional enrichment included genes implicated in the mitotic cell cycle process (strength = 0.82, signal = 0.78, FDR: p = 0.0003). C) Another top cluster consisted of genes connected to piwi, with functional enrichments in cellular process involved in reproduction in multicellular organism (strength = 0.49, signal = 0.45, FDR: p = 0.006), piRNA metabolic process (strength = 1.31, signal = 1.72, FDR: p = 6.97e-08), and egg activation and regulation of oskar mRNA translation (strength = 1.60, signal = 1.62, FDR: p = 9.30e-07). D) The third cluster of interest contains three genes encoding secreted (strength = 0.76, signal = 0.83, FDR: p = 8.01e-05) storage proteins (strength = 1.89, signal = 0.70, FDR: p = 0.004): Lsp2, Lsp1alpha, and Obp99b. All four genes are categorized as hemocyanin/hexamerin (strength = 1.72, signal = 0.92, FDR: p = 0.0006).
(TIFF)

S4 Fig Significant differentially expressed genes, when comparing the interaction between social isolation and sex, are enriched across biological process, local network clustering, and *Drosophila phenotype* terms relating to cell division, reproduction, and hemocyanin/hexamerin proteins. A) Top biological process terms include mitotic cell cycle process and nuclear cell division. B) Top local network cluster terms include egg activation, piRNA metabolic process, and regulation of mitotic cell cycle phase transition. C) Top *Drosophila* phenotypes include abnormal meiotic cell cycle and female sterility.
(TIFF)

S5 Fig Validation of the RNA-seq-derived target genes with qPCR indicates enhanced expression of *takeout* in socially isolated females relative to isolated males. A) A comparison of relative expression fold change values reveals that *takeout* is overexpressed in females but not males following social isolation. The difference in expression between isolated and control flies is significantly greater in females versus males (t(4) = 6.14, p = 0.004). B) Social isolation enhances *Lsp-2* expression in males and females (t(4) = 2.56, p = 0.06). C) Social isolation enhances *Lsp-1α* expression in males and females (t(4) = 2.21, p = 0.09). D) We did not find a statistically significant male-female difference in expression of *Fbp1* following social isolation (t(4) = 1.71, p = 0.16). Data points represent individual cDNA samples for a particular condition relative to a control average. Error bars are ± SEM. **p < 0.01.
(TIFF)

S6 Fig *Takeout* expression is elevated in the brains of females but not males following social isolation. Using relative expression fold change as a measure of the magnitude of change in *takeout* (*to*) expression between the social isolation and control groups, we observed that the values for males are randomly distributed around no change [1], while the female values are largely positive and significantly higher compared to males (t(10) = 2.28, p < 0.05). Data points represent individual cDNA samples for a particular condition relative to a control average. Error bars are ± SEM. *p < 0.05.
(TIFF)

S7 Fig Single cell RNA-seq data from the Fly Cell Atlas identify *takeout* (*to*) expression in neuronal, glial, and epithelial cell types. A) Colors in the ribbon indicate that *to* expression was identified in that cell type through single nucleus RNA sequencing (snRNA-seq) performed by the Fly Cell Atlas project [27]. A redder color on the blue-red gradient indicates that a greater proportion of cells of that particular cell type express *to*. White means that there is no available snRNA-seq data available for that cell type. B) For neurons, *to* was expressed primarily in adult olfactory receptor neurons. C) For epithelial cells, *to* was expressed across numerous cell types of the gastrointestinal tract (crop, midgut, esophagus, and hindgut). D) For glial cells, *to* expression was most widespread within the glia of the adult antennal nerve.
(TIFF)

S8 Fig Knock down of *to* via RNA interference (RNAi) in *to*-expressing cells significantly decreases sucrose preference in isolated females and increases sucrose preference in isolated males relative to their respective controls. A) In ORE-R flies, our preliminary results indicated a female-specific increase in sucrose preference following social isolation (t(15) = 3.14, p = 0.007). B) Flies expressing GAL4 under the control of a *to*-specific promoter do not show differences in sucrose preference following social isolation (t(14) = 0.57, p = 0.58). C) For flies with a UAS enhancer sequence that induces RNAi targeted to *to* mRNA when activated, isolated females increase sucrose consumption relative to control females (t(21) = 3.41, p = 0.003). D) When RNAi targeted to *to* mRNA is induced in *to*-expressing cells, social isolation increases sucrose preference in males (t(14) = 2.31, p = 0.04) yet decreases it in females (t(15) = 5.35, p < 0.0001) relative to respective controls. *p < 0.05, **p < 0.01, ****p < 0.0001.
(TIFF)

S9 Fig Overexpression of *to* in *to*-expressing cells results in no differences in sucrose choice between isolated flies and their respective controls. A) In ORE-R flies, our preliminary results indicated a female-specific increase in sucrose preference following social isolation (t(15) = 3.14, p = 0.007). B) Flies expressing GAL4 under the control of a *to*-specific promoter do not show differences in sucrose preference following social isolation. C) For flies with a UAS enhancer sequence that induces *to* expression when activated, isolated females increase sucrose consumption relative to control females (t(14) = 7.86, p < 0.0001). D) When *to* expression is induced in *to*-expressing cells, social isolation does not alter sucrose preference in males (t(14) = 0.30, p = 0.77) or in females (t(15) = 1.59, p = 0.13) relative to respective controls. **p < 0.01, ****p < 0.0001.
(TIFF)

S10 Fig Knock down of *to* in neurons does not alter sucrose preference across experimental groups. A) In ORE-R flies, females increased their sucrose preference following social isolation (t(15) = 3.14, p = 0.007). B) Flies expressing GAL4 under the control of an *elav*-specific promoter do not show differences in sucrose preference following social isolation in either sex. C) For flies with a UAS enhancer sequence that induces RNAi targeted to *to* mRNA when activated, isolated females increase sucrose consumption relative to control females (t(21) = 3.41, p = 0.003). D) When RNAi targeted to *to* mRNA is induced in neurons, there is no difference in sucrose choice between socially isolated and control flies of either sex. **p < 0.01.
(TIFF)

S11 Fig Overexpression of *to* in neurons does not yield sucrose preference differences between isolated and control flies of either sex. A) In ORE-R flies, females increased their sucrose preference following social isolation (t(15) = 3.14, p = 0.007). B) Flies expressing GAL4 under the control of an *elav*-specific promoter do not show differences in sucrose preference following social isolation in either sex (males: t(16) = 0.30, p = 0.77; females: t(18) = 0.69, p = 0.50). C) For flies with a UAS enhancer sequence that induces *to* expression when activated, isolated females increase sucrose consumption relative to control females (t(14) = 7.86, p < 0.0001). D) When *to* expression is driven in neurons, there is no difference in sucrose choice between socially isolated and control flies of either sex (t(9) = 0.87, p = 0.41). **p < 0.01, ****p < 0.0001.
(TIFF)

S12 Fig Isolated males and isolated females show no differences in sucrose preference relative to controls following knock down of *tra* in neurons. A) In ORE-R flies, sucrose preference is higher in isolated females compared to control females (t(15) = 3.14, p = 0.007). B) Flies expressing GAL4 under the control of an *elav*-specific promoter do not show differences in sucrose preference following social isolation in either sex. C) For flies with a UAS enhancer sequence that induces RNAi targeted to *tra* mRNA when activated, isolated females (t(14) = 2.79, p = 0.01) and isolated males (t(14) = 3.91, p = 0.002) increase sucrose consumption relative to their respective controls. D) When RNAi targeted to *tra* is induced in neurons, there is no difference in sucrose choice between socially isolated and control flies of either sex (males: t(17) = 1.42, p = 0.17; females: t(17) = 0.91, p = 0.38). *p < 0.05, **p < 0.01.
(TIFF)

S13 Fig Downregulation and overexpression of *to* and downregulation of *tra* align with expectations across experimental groups. A) We performed qPCR to confirm genetic knockdown of *to* in the *to*-GAL4 x UAS-*to*^RNAi^ flies and overexpression of *to* in *to*-GAL4 x UAS-*to* flies. *To* overexpression was clearer in control males and control females, which could be due to ORE-R isolated males and isolated females already having relatively high expression of *to*. B) Although the *elav*-GAL4 x UAS-*to* flies showed no change or negative expression of *to* relative to ORE-Rs, the *elav*-GAL4 x UAS-*to*^RNAi^ flies showed downregulation of *to* across experimental groups. C) Genetic knockdown of *tra* in *elav*-GAL4 x UAS-*tra*^RNAi^ flies decreased *tra* expression relative to ORE-Rs. D) Genetic knockdown of *tra* in *elav*-GAL4 x UAS-*tra*^RNAi^ flies also decreased expression of *to* across groups. E) In *elav*-GAL4 x UAS-*to* flies, *to* expression was elevated relative to *elav*-GAL4 flies in control males and isolated males but not in females. F) In *elav*-GAL4 x UAS-*to* flies, *to* expression was elevated relative to UAS-*to* flies across all experimental groups. After completing the VBFD test, flies were frozen on dry ice and decapitated. RNA was extracted from whole fly heads and reverse transcribed into cDNA. All groups represent n = 3 samples/group. Error bars are ± SEM.
(TIFF)

S1 Table Average group size and number of groups tested in the VBFD test for all experimental groups across all genotypes. (XLSX)

S2 Table List of PCR primers used for experiments. (XLSX)

S3 Table Statistics for all 2x2 ANOVAs. (XLSX)

S4 Table Statistics for all one-way ANOVAs. (XLSX)

S1 Data Underlying numerical data for survival analysis. (XLSX)

S2 Data Underlying numerical data for all figures included in the main text. (XLSX)

S3 Data Underlying numerical data for all figures included in Supporting Information. (XLSX)
